# Ionic thermoelectrics: a soft energy conversion technique

**DOI:** 10.1093/nsr/nwag333

**Published:** 2026-06-04

**Authors:** Weishu Liu, Xiaogang Zhang, Nicholas X Fang

**Affiliations:** Department of Materials Science and Engineering, Southern University of Science and Technology, China; Jiangsu Key Laboratory of Electrochemical Energy Storage Technologies, College of Material Science and Technology, Nanjing University of Aeronautics and Astronautics, China; Department of Mechanical Engineering, The University of Hong Kong, China

Converting heat to work has marked several turning points in human civilization. The mastery of fire set humans apart from other primates. The invention of the steam engine propelled human society from an agrarian to an industrial age and also allowed us to enjoy the conveniences of electrification. Thermoelectric technology, based on electrons as energy carriers, has provided the power guarantee for humanity’s deep space exploration. Now, a new class of ionic thermoelectric (i‑TE) materials is emerging, using ions instead of electrons as energy carriers to convert heat to electricity. These materials exhibit exceptionally high (or even giant) thermopower, reaching tens of mV/K, far beyond that of their electronic counterparts [1]. Moreover, their intrinsic softness, stretchability, biocompatibility and low-cost solution processability make them ideal candidates for next-generation wearable power sources that directly harvest human body heat. Other potential applications include self-powering health sensors, thermoelectric effect-induced epidermal theranostics, heat resource recognition for robots, self-powering temperature sensing for fire alarm and other IoT sensors using natural diurnal temperature changes.

The discovery of ‘giant’ ionic Seebeck coefficients (tens of mV/K) is not just a record; it is a physics puzzle that questions our understanding of mass, charge and heat transport in confined, solvated and soft systems. However, the path to application is fraught with paradoxes: high voltage with vanishingly low power and high sensitivity with excruciatingly slow response. Against this backdrop, we have organized this Special Topic on ‘Ionic Thermoelectrics’. Our goal is to bring together cutting-edge research and perspectives from materials chemistry, soft matter physics, electrochemistry and device engineering. We aim to highlight recent breakthroughs that push the boundaries of i–TE performance, deepen mechanism understanding and advance practical applications. This Special Topic does not celebrate a solved problem. Instead, it serves as a call for chemists, physicists and engineers to re‑found the field on rigorous thermodynamics and realistic device physics—transforming a soft curiosity into a hard technology. This Special Topic features a collection of original research articles, short communications, reviews and perspectives contributed by leading groups worldwide.

The long review, led by Lin, Zhang and Liu, provides a bird’s-eye view for future ionic thermoelectrics from ions to products [2]. It systematically reviews recent progress in i‑TE materials and devices, outlines the coming challenges in chemistry and physics, and highlights critical applications that could redefine smart thermal energy utilization. The review also highlights the standardization, scalable manufacturing and reliability that are essential to translating laboratory innovations into viable commercial technologies.

Ionic thermoelectrics is still in its early stages. Accordingly, this Special Topic includes several insightful perspectives on the field’s future. For example, Zhao, Jiang and Crispin directly address the efficiency bottleneck in ionic thermoelectrics based on thermal diffusion (or the Soret effect) of ions, suggesting the need to explore the i–TE materials system with thermal diffusion and thermogalvanic effect, or hybrid systems that leverage both ions and electrons [3]. Yu, Wang and Duan provide a perspective on entropy-engineering of thermopower of i-TE materials with redox couples, which could be one strategy to overcome the efficiency bottleneck [4]. In a different vein, Jiang, Gao and Ma show a totally new perspective on using ionic thermoelectrics for epidermal theranostics [5]. The i-TE epidermal electronics serve dual therapeutic and diagnostic functions, which maximizes the advantage of the high thermopower without concern for efficiency. García-Cañadas proposes an initiative for international cooperation within this small but growing community [6]. Such international cooperation includes establishing regular meetings and conferences to bring together multidisciplinary teams, setting up online courses, tutorials or even summer schools, and standardizing the measurement of materials and devices.

This Special Topic of ‘Ionic Thermoelectrics’ also published three research articles that showcase cutting-edge advances in the field. Pursuing high thermopower is always a hot topic in the TE field. Yue and Zhao report that a hierarchical-structured ionic hydrogel yields a record-high ionic Seebeck coefficient of 71.3 mV/K at minimal temperature gradients (Δ*T* ≤ 2.0 K) [7]. This poly(acrylic acid) wall is perpendicular to the ion transport direction under the temperature gradient, raising a new fundamental question in ion transport. Ma and co-authors have demonstrated 3D digital light processing with a solvent-exchange strategy to fabricate an i–TE device, thereby demonstrating the solution-process advantage of i-TE materials [8]. It would change the paradigm to assemble the full-gel-based i-TE device. Regarding the new application, the unique high thermopower intrinsically corresponds to high sensitivity for thermal sensing or temperature measurement, but it suffers from a slow response due to ion diffusion. Huang and co-authors developed a novel molecular detection method based on the giant i–TE effect in a solid-state ionic polymer [9]. By integrating multiband selective absorption in the fingerprint infrared regimes of target molecules, their approach achieves multi-gas detection capability and extraordinary sensitivity. It fully exploits the high thermopower of i–TE polymers without the typical concerns on the response time. In the short communication, Liu and co-authors have theoretically provided the material figure-of-merit *ZT*_Δ_ on a sound thermodynamic basis, clarifying the puzzle in the field [10].

We thank all authors, reviewers, and the editorial team for their tremendous efforts in making this Special Topic possible. Ionic thermoelectrics is still an emerging field, and many fundamental questions—such as the exact role of water molecules in ionic diffusion or the thermogalvanic reaction of redox couples—remain unanswered. There are also critical applications that could really define the future of i–TE (or thermo-electrochemical) materials and devices. Nevertheless, we believe that this collection of articles provides a timely snapshot of the state of the art and will inspire researchers from diverse disciplines to engage with this exciting soft-energy conversion technique.
